# From Oscillations to Brain States: Real-Time EEG-TMS for Adaptive Neuromodulation

**DOI:** 10.3390/bioengineering13091054

**Published:** 2026-09-10

**Authors:** Melissa Null, Elena Mongiardini, Chiara Leu, Giulia Liberati, Paolo Belardinelli

**Affiliations:** 1 Center for Mind/Brain Sciences—CIMeC, University of Trento, 38068 Rovereto, Italy; melissa.null@unitn.it (M.N.); elena.mongiardini@unitn.it (E.M.); 2 Center for Interdisciplinary Pain Medicine, Department of Neurology and TUM-Neuroimaging Center, School of Medicine and Health, Technical University of Munich, 81675 Munich, Germany; chiara.leu@tum.de; 3 Institute of Neuroscience, Université Catholique de Louvain, 1200 Brussels, Belgium; giulia.liberati@uclouvain.be; 4 Psychological Sciences Research Institute, Université Catholique de Louvain, 1348 Louvain-la-Neuve, Belgium; 5 Department of Neurology & Stroke, University of Tübingen, 72074 Tübingen, Germany; 6 Hertie Institute for Clinical Brain Research, University of Tübingen, 72074 Tübingen, Germany

**Keywords:** transcranial magnetic stimulation, electroencephalography, brain-state-dependent stimulation, closed-loop neuromodulation, neuroplasticity, sensorimotor mu rhythm

## Abstract

Transcranial magnetic stimulation (TMS) enables non-invasive, focal modulation of cortical circuits by inducing electric currents in the brain through electromagnetic induction, thereby influencing neuronal excitability and synaptic plasticity. High inter- and intra-individual variability has led, however, to moderate efficacy and reproducibility of stimulation and treatment protocols, motivating a shift toward brain-state-dependent stimulation. Over the past decade, real-time phase-triggered EEG-TMS has established the oscillatory phase—particularly focusing on the sensorimotor mu rhythm—as a key determinant of cortical excitability and plasticity modulation. The field, however, remains largely confined to univariate, sensor-space analyses of local mu-rhythm phase, missing large-scale network dynamics. Recent advances in online EEG source reconstruction and multivariate machine and deep learning (ML/DL) approaches have begun to move beyond local phase toward whole-brain, network-level state estimation, achieving encouraging preliminary accuracies in predicting trial-by-trial cortical excitability, with promising applications in network-dysregulation conditions such as chronic pain. Integrating source-space reconstruction and individual biological variability, and adaptive ML/DL pipelines into closed-loop frameworks promises to move beyond generic stimulation protocols toward selective, network-targeted neuromodulation tailored to the individual’s dynamic brain state. Against this background, this review provides a critical overview of current achievements and limitations, while highlighting emerging methodological directions toward fully brain-state-adaptive and network-targeted EEG-TMS. We further present an illustrative use case of adaptive EEG-TMS for pain modulation, where treatment responses remain heterogeneous and the relevant dynamics are distributed across networks, and which therefore stands to gain most from individualized, network-targeted protocols.

## 1. Introduction

**Figure 1:**
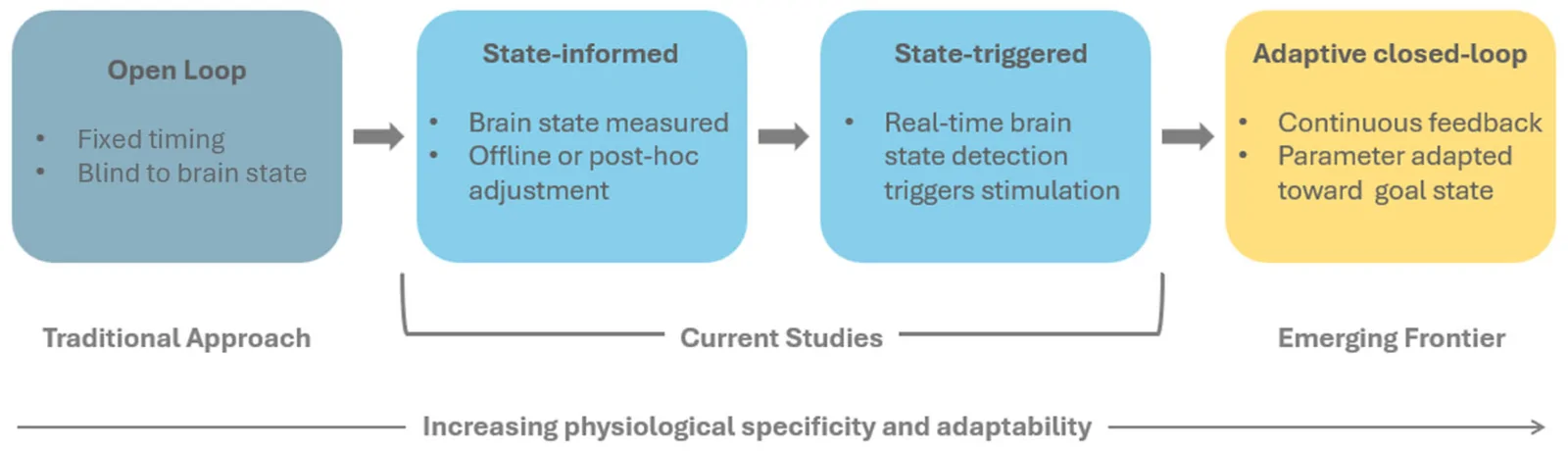
Conceptual overview of the progression of neuromodulation protocols from traditional open-loop stimulation to adaptive closed-loop approaches. The diagram illustrates four stages—open loop, state-informed, state-triggered, and adaptive closed-loop—highlighting increasing physiological specificity and adaptability. As protocols progress, they move from fixed, brain-state-independent stimulation toward continuous feedback systems that dynamically adjust stimulation parameters based on the current brain state to achieve a desired target state.

Non-invasive brain stimulation (NIBS) techniques such as transcranial magnetic stimulation (TMS) and transcranial electrical stimulation (TES) have been shown to modulate cortical activity and induce synaptic plasticity in a brain state-dependent manner [1,2,3,4]. Clinically, repetitive TMS (rTMS) has emerged as a treatment option with minimal side effects for psychiatric diseases such as Major Depression Disorder (MDD) [5,6,7] or obsessive–compulsive disorder (OCD) [8], as well as for patients who have exhausted other available therapies for chronic pain [9]. In the context of brain stimulation, chronic pain is particularly informative because symptoms fluctuate, treatment response is variable, and the relevant neural processes involve distributed networks rather than a single consistently accessible cortical locus [10,11]. However, response rates to rTMS treatment remain modest and variable across individuals [12,13,14]. This variability has been partly attributed to the predefined nature of traditional protocols, which deliver stimulation at fixed intervals regardless of the patient’s fluctuating brain state. Because cortical excitability and network dynamics fluctuate continuously, identical TMS pulses may engage fundamentally different neurophysiological states, leading to variable effects on cortical responses and plasticity. This insight has motivated the development of real-time, brain-state-dependent stimulation paradigms, where brain states are estimated instantaneously to synchronize stimulation with high-excitability phases [1,2,15]. Further, closed-loop EEG-TMS represents a paradigm shift from protocol-driven to brain-state-driven neuromodulation, using real-time neural feedback to adapt stimulation to the ongoing neurophysiological state [16,17,18,19,20] (Figure 1). Alongside hardware and signal processing advances, artificial intelligence and deep learning approaches are increasingly being explored to improve brain-state estimation from high-dimensional EEG signals, representing a complementary frontier for adaptive neuromodulation [17,19]. While progress has been made in demonstrating the effectiveness of brain-state-informed stimulation, current research faces several challenges in defining, measuring, and targeting brain states reliably.

Yet, much less attention has been devoted to a more fundamental question: what constitutes an appropriate representation of the brain state itself? This review addresses this question by critically examining current approaches to brain-state estimation, evaluating their limitations, and discussing how recent methodological advances may support more comprehensive and individualized frameworks for adaptive neuromodulation. First, the concept of brain state and the methods used to measure it will be introduced. Then, the achievements of phase-dependent EEG-TMS in characterizing and modulating cortical excitability and plasticity will be reviewed. The limitations of current state definitions will be examined: relevance in the choice of reference signal, the limits of focusing on local sensor phase, and the challenges of transitioning to source-space approaches. Finally, future perspectives and open challenges on the path toward fully individualized, adaptive neuromodulation will be outlined. Chronic pain is used as a running example of a network-dysregulation condition.

### 1.1. Methods

#### Literature Search and Study Selection

This article is a narrative review and was not designed as a systematic review or meta-analysis. The literature was identified through a targeted search of PubMed and Google Scholar, supplemented by manual screening of the reference lists of relevant reviews and primary research articles to identify additional publications. Searches employed combinations of terms related to transcranial magnetic stimulation (“TMS”, “rTMS”, “theta-burst stimulation”), electroencephalography (“EEG-TMS”, “brain state”, “oscillatory phase”, “mu rhythm”), and adaptive neuromodulation (“brain-state-dependent”, “closed-loop”, “real-time”, “phase-triggered”). Searches were last updated in July 2026.

The search focused primarily on publications appearing between January 2018 and July 2026, while earlier studies were included where necessary to provide methodological or historical context. Study selection was guided by the conceptual aims of the review rather than by exhaustive evidence synthesis. Specifically, publications were included if they contributed to one or more of the following topics: (i) real-time EEG-guided TMS, (ii) brain-state estimation and phase-dependent stimulation, (iii) source-space approaches for EEG analysis, (iv) network-level representations of brain state, or (v) adaptive neuromodulation, including emerging machine learning-based approaches. Methodological papers describing analytical frameworks or software relevant to brain-state estimation were also considered where appropriate.

Conference abstracts were not included unless accompanied by sufficient methodological information, whereas recent preprints were considered only when they addressed rapidly evolving methodological developments directly relevant to the aims of this review and no equivalent peer-reviewed publication was available.

Because the objective of this article is to provide a critical conceptual synthesis rather than a systematic assessment of the literature, no formal risk-of-bias assessment or PRISMA-guided study selection was performed. The publications identified through this process form the basis of the descriptive overview presented in Section 3 (Phase-Triggered EEG-TMS: Key Achievements).

## 2. What Is a ‘Brain State’?

Brain state is best regarded as an operational construct describing the instantaneous configuration of neural activity, whose characterization depends on the spatial, temporal, and physiological information accessible to the measurement modality. Different neuroimaging and neurophysiological techniques probe neural activity at distinct spatial and temporal scales and capture different aspects of the underlying physiology, yielding complementary but necessarily incomplete representations of the brain’s ongoing state. For example, electroencephalography (EEG) and magnetoencephalography (MEG) provide millisecond-resolution measurements of electrophysiological activity and naturally lend themselves to defining brain states in terms of instantaneous oscillatory phase, amplitude, spectral power, or inter-regional synchrony. In contrast, functional magnetic resonance imaging (fMRI) characterizes brain states through slow fluctuations of the blood oxygenation level-dependent (BOLD) signal and large-scale functional network configurations evolving over seconds, whereas positron emission tomography (PET) reflects even slower changes in metabolic activity or neurotransmitter dynamics on timescales of minutes to tens of minutes. Consequently, what is referred to as a “brain state” may represent transient electrophysiological excitability, oscillatory coupling as a metric of inter-areal connectivity, hemodynamic network configuration, or metabolic condition, depending on the acquisition modality and analytical framework. This diversity is particularly relevant for closed-loop neuromodulation, where the definition of the monitored state—ranging from membrane potential fluctuations [21] to large-scale dynamic functional connectivity [22]—determines not only the measurable variables but also the temporal precision and physiological mechanisms through which stimulation can be synchronized with ongoing brain activity [23]. While EEG captures only an incomplete but temporally precise representation of the brain’s underlying state, its millisecond-scale temporal resolution makes it one of the most suitable modalities for real-time state estimation and closed-loop neuromodulation, where rapid detection and adaptation are essential.

Neurophysiologically, some processes are mainly confined to a single area [24] while others involve relevant coordinated interaction between multiple regions forming cortical networks [25]. These spatially restricted activity patterns suggest that brain states can be defined at a local scale, spanning just a few centimeters of cortical tissue as in the case of the sensorimotor hand knob [26]. Temporally, brain states based on electrophysiological metrics are structured through endogenous rhythms which synchronize neuronal populations across functional networks both during rest and task [27]. Critically, different phases of a rhythm generate transient time-windows of enhanced excitability [1,28], as shown in studies examining the dynamics of mu-alpha and beta rhythms of the sensorimotor cortex [1,29,30,31,32]. These endogenous oscillations (Box 1) span a broad frequency range, with distinct brain regions exhibiting characteristic oscillatory frequencies that are further shaped by ongoing sensory, cognitive, and behavioral demands [33].

To bridge these diverse perspectives, brain states have recently been conceptualized as dynamic configurations of neural activity and functional coupling that emerge from specific physiological and cognitive conditions and shape ongoing physiological processes and behavior. These criteria encompass both spatial and temporal dimensions and unify descriptions at micro- and macro-level [23,34].

This review focuses on endogenous neuronal oscillations measured with EEG and their influence on TMS responsiveness, with particular emphasis on oscillatory phase as a target for real-time, brain-state-dependent stimulation. Rather than merely brain-state correlates, neuronal oscillations represent a key dynamical mechanism through which large-scale neural activity is organized across time [35,36]. Their ongoing fluctuations shape moment-to-moment variations in cortical excitability and network communication, providing a physiologically grounded substrate for defining EEG-based brain states. Consequently, tracking oscillatory phase in real time offers the possibility of delivering stimulation at precisely those moments when cortical circuits are most susceptible to external perturbation, thereby improving the specificity and effectiveness of neuromodulation [1].

Box 1 **Physiological basis of endogenous cortical oscillations.**

Endogenous neuronal oscillations arise across brain regions in animals and humans, during both resting and task-engaged conditions [27]. Their physiological role is closely linked to the synchronization of neuronal activity within functionally coherent but spatially distributed networks. Oscillatory power at the macroscopic level reflects collective large-scale synchronization of neuronal assemblies. This synchronization defines momentary windows of excitability that are a fundamental property not only of sensorimotor systems [1,27,31,37,38] but also of the prefrontal and sensory networks that govern cognitive and perceptual functions [3,15,17]. Microscopically, rhythmicity emerges from the dynamic interplay of multiple cell types, including excitatory pyramidal neurons and diverse classes of inhibitory interneurons, whose coordinated activity generates oscillatory cycles across a wide frequency range (<1 Hz to >100 Hz); the precise cellular mechanisms differ across frequency bands and brain regions [33]. Canonical frequency bands include delta (<4 Hz), theta (4–8 Hz), alpha (8–13 Hz), beta (13–30 Hz); while these boundaries are operationally useful, the functional roles of individual bands are context-dependent and not strictly separable [39]. The rhythmic field potentials captured by MEG and EEG primarily reflect the summed postsynaptic currents of cortical pyramidal neurons, particularly those with apical dendrites, oriented perpendicular to the cortical surface, spanning multiple cortical layers [40]. First proposed in the visual system, the idea that distinct phases of cortical oscillations correspond to windows of heightened or reduced excitability has since been extended to other systems, effectively framing oscillatory phase as a fundamental mechanism gating information flow and cortical responsiveness [28].

## 3. Phase-Triggered EEG-TMS: Key Achievements

Over the past decade, phase-triggered EEG-TMS has evolved from methodological proof of concept to a more established experimental framework for probing and manipulating brain-state-dependent neural dynamics. Collectively, the associated studies demonstrate that the brain’s responsiveness to external stimulation is not static but fluctuates systematically according to the temporal dynamics of endogenous neuronal rhythms. As summarized in Table 1, phase-dependent effects have been observed across a range of experimental paradigms and outcome measures. Notably, the vast majority of included studies employed state-triggered designs, in which stimulation is delivered upon real-time detection of a predefined oscillatory phase; to the best of our knowledge, no published study has yet used real-time connectivity measures to directly trigger TMS pulses, with Vetter et al. [41] representing the closest approach to a connectivity-informed triggering strategy. For clarity, the findings reviewed in this section are organized into two broad categories: (i) single-pulse EEG-TMS studies, which primarily probe phase-dependent fluctuations in instantaneous cortical excitability, and (ii) repetitive stimulation protocols, which leverage oscillatory phase to determine magnitude and direction of longer-lasting neuroplastic changes.

### 3.1. Single-Pulse Findings: Phase as a Readout of Instantaneous Excitability

**Figure 2:**
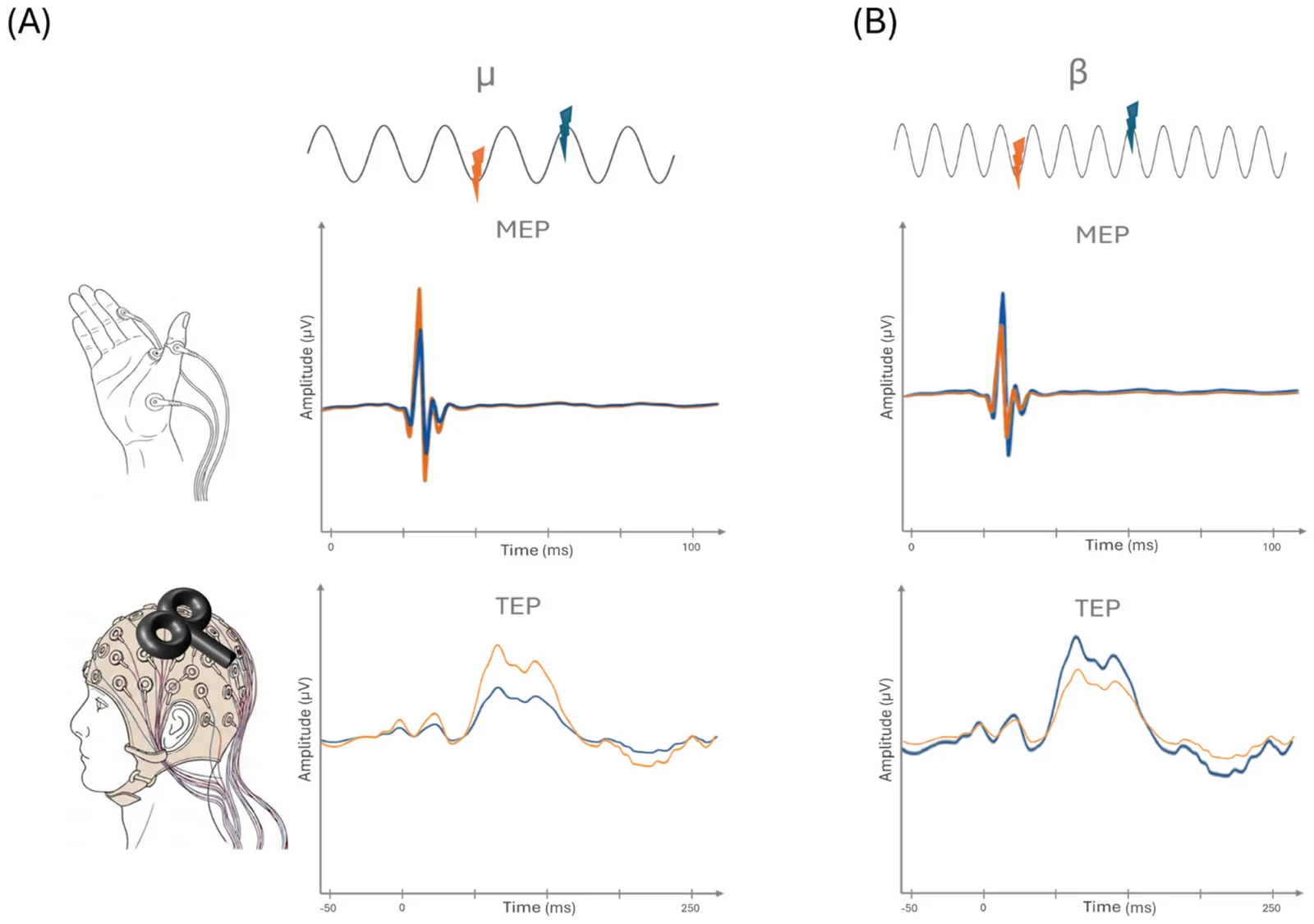
Effects of EEG–spTMS targeting M1. Real-time EEG is used to track local oscillatory phase (peak/trough of mu or beta oscillation) to trigger single TMS pulses. Phase-dependent excitability is quantified via two measures: TEPs (TMS-Evoked Potentials), reflecting cortico-cortical excitability, and MEPs (Motor-Evoked Potentials) recorded from the hand, reflecting corticospinal excitability. The waveforms shown represent the grand average TEP across all channels. (**A**) Sensorimotor Mu Rhythm: For the sensorimotor mu rhythm (~8–13 Hz), the trough (orange) represents the window of highest excitability. Delivering a single TMS pulse at the mu trough results in significantly higher MEP amplitudes and larger TEP component amplitudes compared to stimulation delivered at the positive peak (blue), which typically decreases measures of excitability [1,42,48]. (**B**) Beta Oscillation: The local beta oscillation (13–30 Hz) exhibits the opposite phase-dependency. The phase of highest excitability is inverted: MEP and early TEP amplitudes are largest when the single TMS pulse is delivered at the positive peak and falling phase (blue) rather than the trough (orange) [44,48,53].

The most robust and consistently replicated finding is that single TMS pulses delivered to the primary motor cortex (M1) during the trough of the individual sensorimotor mu rhythm (~8–13 Hz) elicit larger motor-evoked potentials (MEPs). Phase is typically estimated from a C3 Hjorth filter—a Laplacian filter enhancing the local cortical signal by weighting the central electrode against its neighbors. Stimulation at the trough consistently yields a lowered resting motor threshold (RMT; the minimum TMS intensity required to elicit a reliable motor response at rest) and larger motor-evoked potentials (MEP) compared to stimulation at random phase [42,43,44,45,48,52,53] (Figure 2A). Notably, the phase of maximum effect may not be exactly the trough but a slightly later phase, with the optimal timing varying across and within sessions and subjects [46,47,48,49,50,51,52,53,54,55]. Stimulation at the positive peak has been shown to typically decrease measures of cortical excitability [43,45,46,47,48,53]. However, other real-time EEG-triggered TMS studies targeting the sensorimotor mu-rhythm have failed to observe reliable positive peak modulation of corticospinal excitability [1,2].

Furthermore, it has been observed that in healthy individuals the phase-dependency of the RMT is symmetrical across hemispheres, suggesting that it is not tied to motor dominance or specific anatomic lateralization but instead a physiological trait of the human motor system [42,43].

This effect is specific to the oscillation in question: while in case of the mu rhythm the trough constitutes the phase of highest cortical excitability, the opposite has been found for the beta oscillation (13–30 Hz) in M1, where MEPs have been found largest at the positive peak and falling phase of the local beta oscillation [43,45,48,53]. Not only does delivering single TMS pulses at the optimal beta phase result in larger MEPs but it also increases the consistency and speed with which MEPs are generated [44,48] (Figure 2B). Despite growing interest in beta-phase-dependent stimulation, the extent to which sensorimotor beta oscillations represent an independent mechanism of excitability modulation, rather than a harmonic expression of the mu-alpha rhythm, remains to be established.

These findings position the sensorimotor mu rhythm as a robust physiological determinant of excitability, where the oscillation trough represents a highly consistent window of maximum corticospinal excitability which appears to be independent of hemispheric dominance [43].

While MEPs are a reliable, albeit indirect, measure of corticospinal excitability, they are inherently limited to the sensorimotor system. TMS-evoked potentials (TEPs)—electroencephalographic responses phase-locked to a TMS pulse—extend this picture by capturing both local cortical excitability and the subsequent spread of activity to distant regions. Their components, operationally defined by polarity (P, positive; N, negative) and latency (e.g., N100, negative peak at 100 ms), reflect specific physiological mechanisms, with early peaks typically linked to excitatory responses and later components (in particular N45) associated with inhibitory neurotransmission [56,57,58,59]. Mirroring the MEP findings, phase-dependent effects extend to TEPs: single pulses delivered at the trough versus the positive peak of the sensorimotor mu rhythm produce significantly larger P70 and N100 amplitudes at 110% RMT, with the N100 effect persisting at 90% RMT [42]. Notably, this phase-dependency of TEP components extends to subthreshold stimulation intensities, where no MEP is elicited, confirming that the mu trough reflects a state of heightened cortical rather than purely corticospinal excitability [60]. Both mu and beta rhythm have been found to modulate early TEP components (specifically P50-N15) whereas the late component (N100-P50) is modulated by the mu rhythm alone [50]. Importantly, the phase-dependency of TEPs is often abolished in the affected hemisphere of stroke patients [51,52] which has the potential to serve as a specific biomarker for pathological brain network dysfunction.

At the network level, stimulation delivered at the mu trough has been reported to strengthen interhemispheric theta- and alpha-band phase-locking after TMS [47], indicating that phase-dependent stimulation can modify large-scale network interactions. Conversely, the network configuration preceding stimulation also influences TMS efficacy: stronger pre-stimulation interhemispheric functional connectivity between the primary motor cortices predicts greater corticospinal excitability independently of local oscillatory phase [36,41]. Thus, functional connectivity appears to play a dual role, serving both as a target of and a determinant of brain-state-dependent neuromodulation.

### 3.2. EEG-rTMS Findings: Phase as a Determinant of Plasticity Direction

Whereas single-pulse protocols reveal the instantaneous relationship between oscillatory phase and cortical excitability, repetitive TMS protocols employ this relationship to drive lasting changes in synaptic efficacy. The direction and magnitude of these plastic changes—whether long-term potentiation (LTP)-like or long-term depression (LTD)-like—are not determined by stimulation frequency itself, but emerge from an interaction between the repeated TMS impulses and the instantaneous brain state at the moment of delivery.

**Figure 3:**
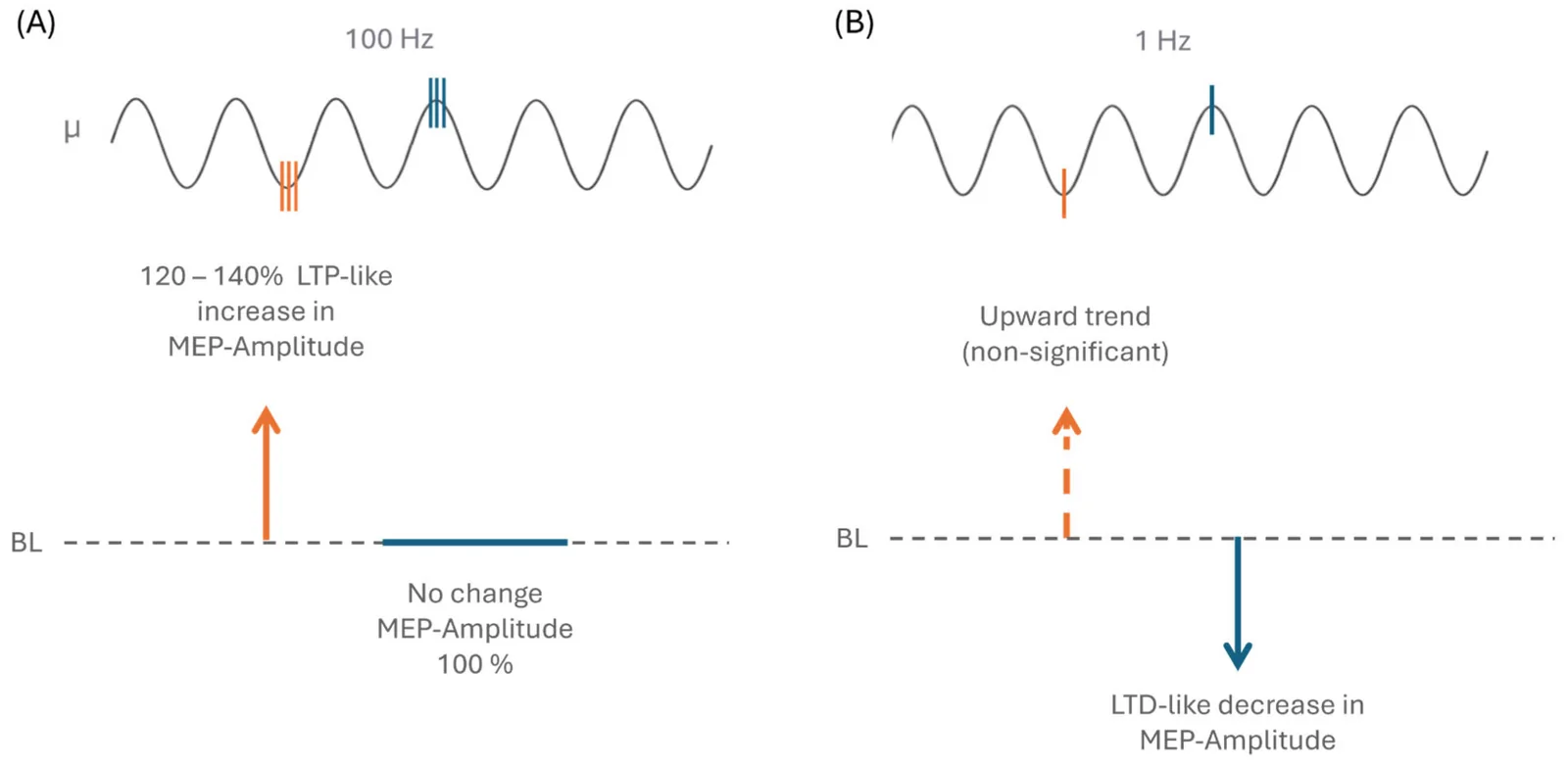
Phase-dependent modulation of plasticity direction in EEG-rTMS. (**A**) High-frequency (100 Hz) burst stimulation: Delivering high-frequency bursts (such as triplets or quadruplets) synchronized to the mu trough (orange bars) induces LTP-like plasticity, resulting in an increase in MEP amplitude (up to 120–140% relative to baseline). Conversely, stimulation at the positive peak (blue bars) fails to enhance excitability, maintaining baseline levels (100%). (**B**) Low-Frequency (1 Hz) stimulation: Applying single TMS pulses repeated at 1 Hz at the positive peak (blue) leads to a LTD-like reduction in MEP amplitude. Applying the same single-pulse 1 Hz protocol at the mu trough (orange) shifts the effect toward an upward trend indicative of LTP-like plasticity. To date, reported plasticity effects have been observed to persist at least 45 min [1,2,3].

rTMS protocols delivering high-frequency (100 Hz) triplet [1] and quadruplet [46] bursts synchronized to the mu trough successfully induced LTP-like increases in MEP amplitude, demonstrating that the fluctuations of excitability can be instrumental to enhance the impact of existing clinical rTMS protocols (Figure 3A). In contrast, low-frequency rTMS timed to the positive peak of the mu rhythm results in LTD-like reduction in MEP amplitude (Figure 3B). Delivering the same low-frequency protocol at the trough shifted the effect toward a trend for LTP-like plasticity, confirming that the phase of the mu rhythm determines the direction of the plastic changes [2].

**Figure 4:**
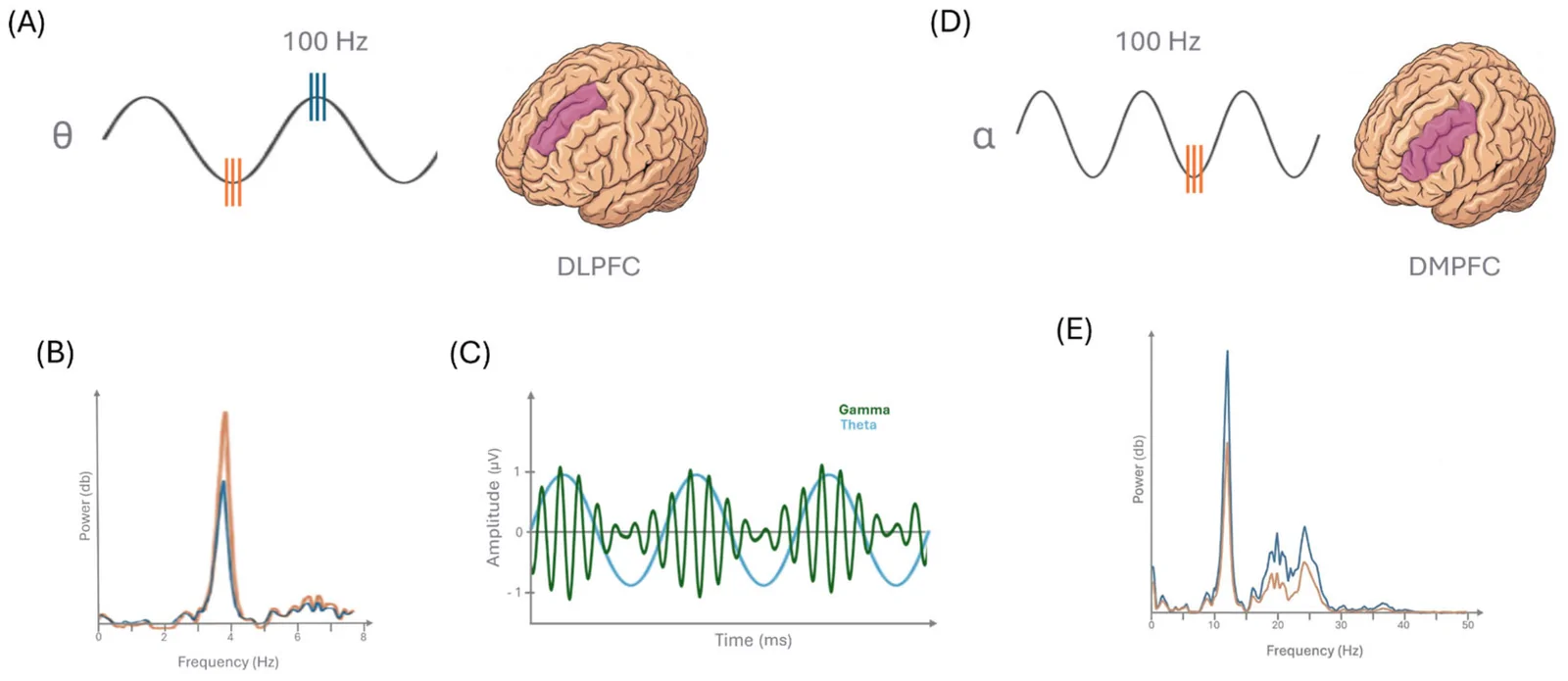
Phase-dependent prefrontal stimulation. High-frequency 100 Hz triplet stimulation of DMPFC synchronized to specific phases of the local theta oscillation (**A**). Synchronizing the high-frequency triplet bursts to the trough of the theta oscillation significantly increases TMS-induced theta power (orange line) compared to peak-targeted stimulation (blue line) (**B**). Theta-trough stimulation leads to increased phase–amplitude coupling of prefrontal theta and posterior gamma oscillations (**C**) and increased performance in a working memory task. High-frequency 100 Hz triplet stimulation of DLPFC synchronized to the prefrontal alpha rhythm (orange bursts) (**D**) leading to reduced resting-state alpha power (orange) compared to pre-stimulation resting-state alpha power (blue) (**E**).

These findings establish a basis for translating brain-state-dependent rTMS into clinical practice, where synchronized protocols have since been tested across neurological and psychiatric conditions with promising results. Repetitive 100 Hz triple-pulse rTMS synchronized to the ipsilesional mu trough provided significant clinical improvements in motor impairment and motor function in chronic stroke patients, showing effects comparable to established 1 Hz protocols [49]. Beyond motor rehabilitation, brain-state-dependent protocols have been extended to prefrontal targets. Synchronizing high-frequency triplet bursts to the negative peak of theta oscillations in the dorsomedial prefrontal cortex (DMPFC) increased TMS-induced theta power and significantly reduced working memory response times [3] (Figure 4A–C). Moreover, in patients with major depressive disorder, alpha-synchronized triplets delivered to the left dorsolateral prefrontal cortex (DLPFC) reduced resting-state alpha power and increased TMS-induced beta oscillations. Both effects were absent with standard non-synchronized protocols [45] (Figure 4D,E).

Lastly, administering dual-coil paired pulses bilaterally showed that short-interval interhemispheric inhibition (SIHI)—a marker of effective inhibitory interhemispheric motor connectivity—is strongest when both motor cortices are stimulated while in-phase at the negative peak of the mu rhythm [35]. This indicates that effective interhemispheric communication depends not only on anatomical connectivity but on the momentary temporal alignment of local rhythms across the motor network.

Taken together, these findings demonstrate that synchronizing TMS to the instantaneous oscillatory brain state reliably modulates cortical excitability, shapes the direction of plasticity, and, in selected studies, improves physiological and behavioral outcomes. Collectively, these studies establish oscillatory phase as a physiologically meaningful variable that can be harnessed to guide TMS in real time. At the same time, they raise broader questions about how brain states should be defined for brain-state-dependent neuromodulation.

Local oscillatory phase can therefore be regarded as the first experimentally validated marker of cortical responsiveness to TMS. This does not imply, however, that it provides a complete description of the underlying brain state. Rather, these findings raise the broader question of which neural features—or combinations of features—most faithfully capture the momentary responsiveness of cortical networks.

## 4. Open Questions: The Limits of Current ‘Brain State’ Definition

**Figure 5:**
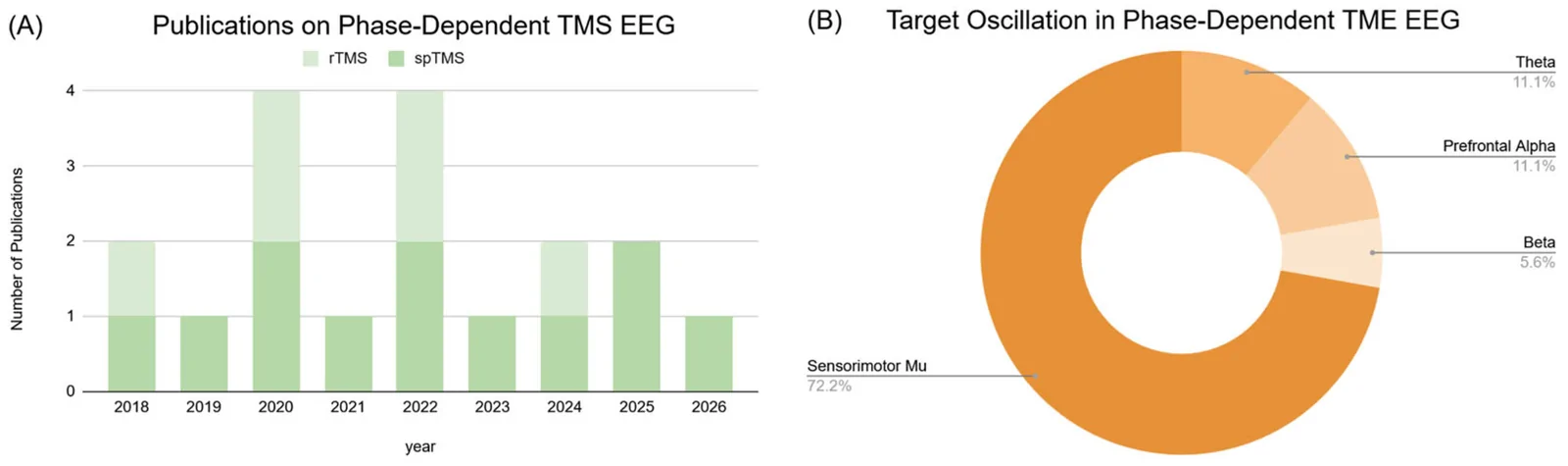
Publication trends and oscillatory targets in phase-dependent TMS-EEG research. (**A**) Publications on Phase-Dependent TMS-EEG (2018–2026). Bar chart depicting the annual number of publications employing phase-dependent EEG informed TMS, stratified by stimulation type: repetitive TMS (rTMS; dark green) and single-pulse TMS (spTMS; light green). Publication output increased notably from 2018, peaking in 2021–2022, and has remained modest through 2026, with spTMS representing the predominant paradigm across most years. (**B**) Target Oscillation in Phase-Dependent TMS-EEG Publications included in this review. Donut chart illustrating the distribution of neural oscillations targeted across the identified publications. Sensorimotor Mu rhythm constitutes the largest proportion (72.2%), followed by Theta (11.1%), Prefrontal Alpha (11.1%), and Beta (5.6%), reflecting a strong methodological focus on sensorimotor oscillations in the existing literature.

Despite the progress summarized above, phase-dependent EEG-TMS studies are still relatively scarce and have been conducted within a remarkably homogeneous methodological framework, with most investigations relying on similar recording modalities, stimulation targets, oscillatory features, and outcome measures. Consequently, our current understanding of brain-state-dependent stimulation is largely derived from a restricted experimental landscape, leaving many fundamental questions regarding the definition, measurement, and exploitation of brain states unresolved. This is most clearly illustrated by the overwhelming emphasis on a single oscillatory target—the sensorimotor mu rhythm—which accounts for nearly three quarters of all published studies (Figure 5). The sensorimotor mu rhythm has provided an ideal initial target for brain-state-dependent stimulation because it combines a high signal-to-noise ratio with a well-defined spatial topology, while the motor hand knob offers one of the most extensively characterized cortical targets and an objective physiological readout through MEPs. These characteristics have enabled rapid methodological progress but have also shaped the field around a highly favorable experimental model. Whether the same principles generalize to other oscillatory rhythms, cortical regions, or large-scale brain networks—where oscillatory signatures are often weaker, spatially distributed, and functionally more heterogeneous—remains largely unknown. This concern is not merely theoretical. The specific properties that make the sensorimotor mu rhythm an ideal target—a single accessible generator, a high signal-to-noise oscillation, and an objective peripheral readout in the MEP—are precisely those absent in the conditions which neuromodulation is most often called upon to treat. Chronic pain is the clearest example: there is no single cortical locus [10,61], no peripheral analogue of the MEP, and the clinical target is defined by distributed network reorganization rather than by the excitability of a single cortical patch [62,63,64].

A second, largely independent limitation concerns the stimulation protocols themselves. Even when stimulation is delivered according to the current brain state, most neuromodulation paradigms still rely on stimulation parameters (e.g., frequency, intensity, pulse number, or burst pattern) that are selected *a priori* and remain fixed throughout the intervention. Recent evidence further questions this conventional strategy. A large-scale meta-analysis of more than 200 rTMS studies showed that the traditional binary distinction between “excitatory” and “inhibitory” protocols is overly simplistic, with smaller-than-expected effect sizes, poor test–retest reproducibility, and inconsistent modulation of TMS-evoked potentials [14]. These findings suggest that variability arises not only from differences in the brain state at the time of stimulation but also from the assumption that a single predefined stimulation protocol can produce consistent effects across individuals and sessions.

An additional source of variability that has received comparatively little attention in the neuromodulation literature is hormonal state. Growing evidence indicates that the efficacy of plasticity-inducing protocols varies across menstrual cycle phases [65,66,67]. Unlike the instantaneous electrophysiological features targeted by real-time pulse triggering, hormonal state operates over timescales of weeks and is therefore not a candidate for closed-loop integration. It is nonetheless a systematic source of inter-individual and intra-individual variability that current studies rarely control for, and whose neglect likely contributes to the poor reproducibility of plasticity protocols across individuals and sessions. The following sections examine these limitations in greater detail, focusing on the definition of the EEG reference signal, the sufficiency of local oscillatory phase as a brain-state descriptor, and the transition toward source-space representations.

### 4.1. Selecting an Appropriate Reference Signal to Define the Target State

Importantly, there is no unique “true” EEG oscillatory phase that can be unambiguously assigned to the activity of a specific cortical region. Rather, every phase estimate depends on how the underlying neural activity is represented, whether through sensor-level recordings or source reconstruction. Consequently, the target brain state in EEG-guided neuromodulation is inseparable from the choice of the reference signal. This choice is informed by anatomical, physiological, and technical considerations, but it remains an operational approximation rather than a uniquely defined representation of the brain’s ongoing state.

A weighted combination of scalp sensors as the Hjorth filter around C3 for the sensorimotor cortex [1] or around FC3 for the left dorsolateral prefrontal cortex [45] may offer improved sensitivity to the activity of interest, but the choice of the weighting scheme is neither unique nor physiologically neutral. Each spatial filter defines a different projection of the underlying electromagnetic field and therefore a different representation of the putative brain state [1,44,68,69]. Consequently, phase-dependent stimulation studies may appear to target the same oscillation while in practice operating on distinct signal representations, complicating comparisons across studies and raising the possibility that discrepancies in the literature reflect differences in state definition rather than differences in the underlying neurophysiology [70].

**Figure 6:**
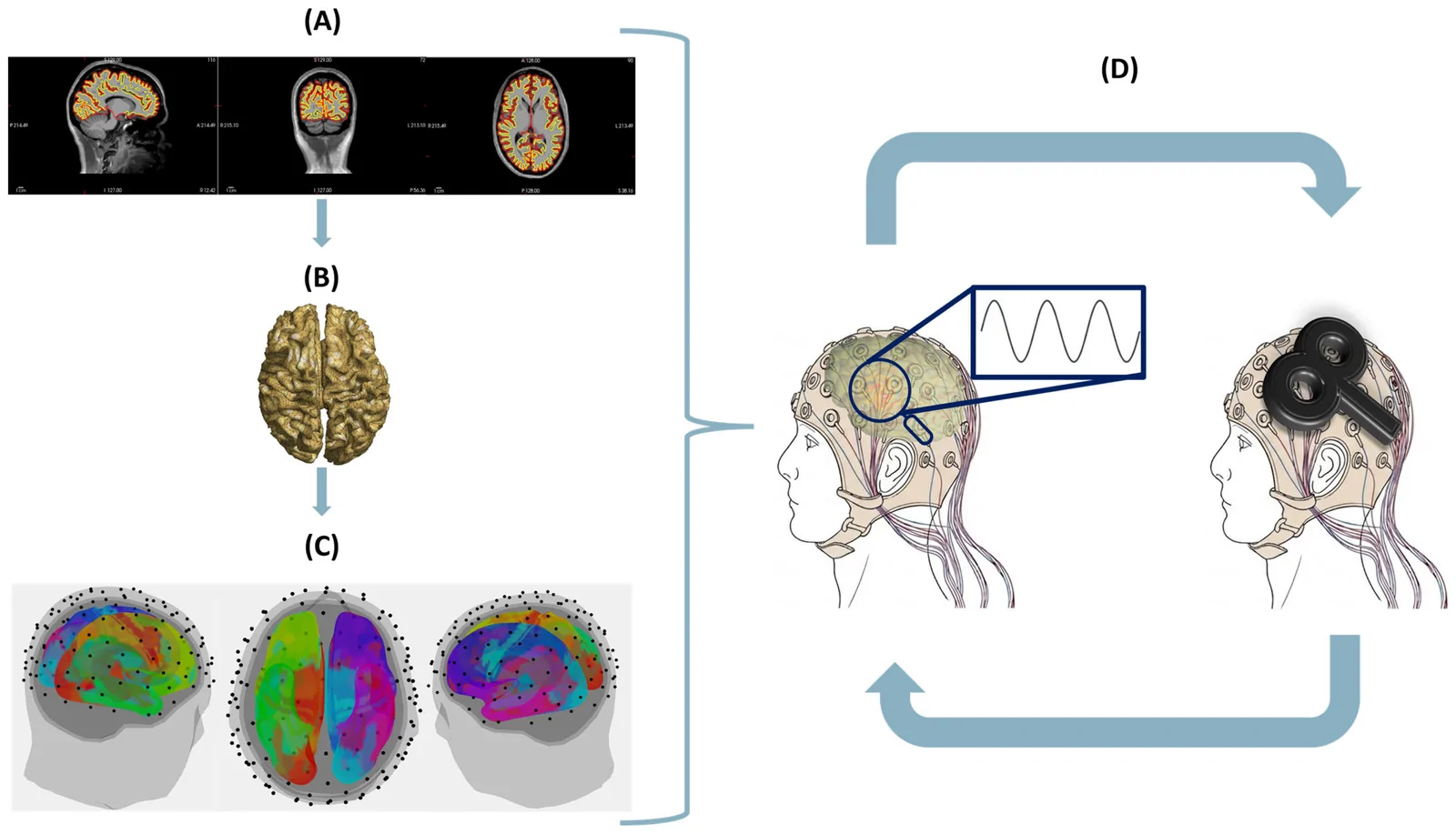
Pipeline for individualized online EEG source reconstruction and brain-state-dependent TMS. (**A**) Quality control of white matter (yellow) and grey matter (red) boundary segmentation derived from structural MRI; manual correction may be required where automated segmentation fails. (**B**) Individual cortical mesh generated by averaging white and gray matter meshes and resampling to a standard number of points. (**C**) Co-registration of EEG electrode positions (black dots) with the individual cortical parcellation defining the source space for inverse modelling. The parcellation shown represents the Glasser Atlas (360 parcels) [77]. (**D**) Closed-loop paradigm: continuous EEG is projected into source space and instantaneous phase is extracted from the target region in real time. When the predefined brain-state criterion is met, a TMS pulse is triggered and the neural response is fed back into the monitoring pipeline, completing the loop. It should be emphasised that steps A–C are performed offline prior to the EEG-TMS real-time experiment. Leadfield calculation can be executed upon recording of individual EEG electrode positions on the head of the subject.

Indeed, a growing body of evidence suggests that the central challenge in phase-triggered EEG-TMS may not be identifying the correct phase, but establishing whether a stable phase–excitability relationship exists in the first place. While population-level effects are reproducible, individual-level effects are sometimes unstable and poorly reproducible across sessions [71]. These observations raise the possibility that the mu-rhythm phase measured from a given EEG channel combination is unlikely to reflect a single stationary physiological process, but rather a dynamic mixture of oscillatory generators whose relationship to corticospinal excitability evolves over time. Under this view, inconsistencies across studies may reflect not only methodological differences but also the intrinsically non-stationary nature of the brain states being targeted. Source reconstruction (Figure 6; see Box 2 for implementation details) may improve anatomical specificity and reduce signal mixing, although it does not eliminate the fundamental dependence of the inferred state on the underlying spatial model. Nevertheless, combining source reconstruction with individualized anatomical and functional information, together with atlas-based parcellation, may provide a more physiologically meaningful definition of the target state [72]. These considerations motivate investigation of whether individualized, model-based representations of brain activity can provide a more informative basis for brain-state estimation than fixed sensor-level biomarkers.

Box 2 **Online EEG source reconstruction for brain-state-dependent neuromodulation.**

Real-time source reconstruction enables closed-loop neuromodulation to be driven by cortical activity estimated in source rather than sensor space, thereby improving anatomical specificity and reducing the influence of spatial mixing inherent to scalp EEG. A typical implementation relies on an individualized structural MRI to construct a high-resolution (>10 K mesh points) cortical source model and realistic head volume conductor, combined with high-density EEG and session-specific digitization of electrode positions. The cortical surface is commonly generated from FreeSurfer-derived white and pial surfaces, from which a midthickness mesh is obtained as their geometrical average and decimated while preserving inter-subject correspondence [73,74]. Boundary meshes for scalp, skull, and brain are then generated to compute a forward model [75] and the corresponding leadfield matrix, which describes the contribution of each unitary cortical dipole to the scalp potential distribution. Because electrode positions vary across recording sessions, leadfields must generally be recalculated after sensor co-registration to the individual anatomy. The key steps of this pipeline—from MRI-based segmentation and surface reconstruction (Figure 6A,B), to electrode co-registration and cortical parcellation (Figure 6C), and finally the resulting closed-loop stimulation framework (Figure 6D)—are illustrated in Figure 6.

Source reconstruction is performed by applying an inverse operator to the EEG data, typically using adaptive spatial filters such as linearly constrained minimum variance (LCMV) beamformers [76]. In the real-time practice, the beamformer weights are computed offline from a covariance matrix estimated on calibration data acquired under conditions closely matching the subsequent real-time experiment, ensuring that the statistical properties of the signals of interest are appropriately represented. Once computed, the spatial filter can be applied online with limited computational overhead compatible with real-time operation, yielding continuous source time series from a priori anatomically or functionally defined cortical regions or parcels. Rather than reconstructing activity exclusively from a single cortical location, LCMV beamformers construct spatial filters that maximize sensitivity to activity originating from the target region while attenuating contributions from competing sources. Differently, distributed inverse methods such as Minimum Norm Estimation (MNE) reconstruct activity across the entire source space. Nevertheless, both LCMV and MNE can support efficient online estimation once the computationally intensive forward modelling and inverse operator computations have been performed offline. The reconstructed source signals can then be used to estimate oscillatory phase, amplitude, power, or inter-regional functional connectivity, providing the physiological variables that guide stimulation.

While the requirement for individual MRI, accurate electrode localization, and high-density recordings increases methodological complexity, it enables anatomically constrained source estimates with substantially improved spatial specificity and source separation compared with sensor-space analyses, providing a principled framework for individualized closed-loop stimulation. However, the effective spatial accuracy of source reconstruction remains dependent on several factors, including the quality of the anatomical head model, tissue conductivity assumptions, electrode co-registration, preprocessing strategy, inverse modelling approach, and the signal-to-noise ratio of the EEG recordings [72]. Additional limitations include the reduced detectability of deep sources and, for adaptive inverse methods such as LCMV, the dependence on robust covariance estimation.

Although online source reconstruction is increasingly feasible, its performance should not be interpreted as independent of these modelling assumptions. Rather, the fidelity of real-time source estimates reflects the quality of the offline forward model, inverse solution, preprocessing pipeline, and signal quality. Consequently, source reconstruction should be regarded as an anatomically and physiologically informed estimate of ongoing cortical activity rather than a direct measurement. Importantly, because the computationally demanding modelling steps—including head modelling, leadfield computation, source-space definition, cortical parcellation, and inverse operator estimation—can be performed offline, individualized source imaging remains compatible with the temporal constraints of closed-loop EEG-TMS and represents a promising strategy for improving the spatial specificity of brain-state estimation.

### 4.2. Beyond Local Phase

#### 4.2.1. Network State Evidence

Recent EEG-TMS studies suggest that the functional impact of stimulation is shaped not only by the local oscillatory phase but also by the instantaneous connectivity state of the underlying network. Stefanou et al. [35] demonstrated that interhemispheric communication between the two motor cortices depends on the phase relationship of ongoing sensorimotor mu-rhythms, with transcallosal interactions varying according to the degree of phase synchrony between the hemispheres. This finding provided direct evidence that effective connectivity fluctuates dynamically as a function of ongoing oscillatory network states. Subsequently, Vetter et al. [41] showed that corticospinal excitability can be predicted from real-time EEG connectivity measures, with TMS delivered during highly connected motor-network states producing larger MEPs than stimulation delivered during weakly connected states. Together, these studies suggest that network interactions provide information about cortical responsiveness beyond that contained in local oscillatory activity alone. More recently, Ermolova et al. [78] extended this framework to plasticity induction using source connectivity-dependent cortico-cortical paired associative stimulation (ccPAS). While phase-specific ccPAS produced robust changes in both effective and functional interhemispheric connectivity, these effects were largely independent of the targeted phase relationship between the two motor cortices and were also observed during random-phase stimulation, raising questions about the extent to which connectivity plasticity can be optimized through simple phase-based state definitions.

#### 4.2.2. Predictive Accuracy Leveraging Whole Cortical States

Recent studies have moved beyond handcrafted, single-channel EEG biomarkers by applying machine learning (ML) and deep learning (DL) approaches directly to high-dimensional EEG signals. Individualized spatio-temporal patterns have been shown to decode trial-by-trial fluctuations in both corticospinal excitability [79] and specific TEP amplitudes, such as the N45 component tracked by the PRIME framework ([17,80]; see Box 3 for details). These findings suggest that stimulation-relevant brain states may be represented by distributed multivariate neural signatures extending beyond conventional oscillatory phase or power biomarkers.

Although performed with intracranial electrical stimulation rather than TMS, recent work by Rabuffo et al. provides important conceptual support for whole brain-state-dependent neuromodulation [15]. Across both stereo-EEG and high-density EEG, multiple features of spontaneous neural activity predicted the magnitude and structure of subsequent stimulation responses, indicating that response variability reflects structured fluctuations in ongoing brain dynamics rather than random noise. Notably, distributed network-level descriptions of the pre-stimulus state explained substantially more variance than local activity alone, suggesting that stimulation responsiveness emerges from large-scale brain configurations rather than the state of a single cortical region. Together, these findings support a shift from narrowly defined phase-based approaches toward broader, multidimensional characterizations of brain state—the unresolved challenges and validation requirements of which are summarized in Table 2.

Box 3 **Machine learning and deep learning methods for brain-state predictive modeling.**

The studies discussed in the present review illustrate how phase-based triggering strategies have provided a first approximation of the brain state. However, the growing body of research around the concept of “brain state” [23,36] suggests that this extends beyond the oscillatory phase of a single channel, encompassing amplitudes and spectral features across multiple frequency bands, network-level connectivity measures, and an increasing number of controllable TMS parameters. This shift transforms brain-state estimation from a univariate, threshold-based problem into an intrinsically multidimensional one. In this context, ML and DL offer a possible solution, as they can integrate heterogeneous, high-dimensional feature domains, capture their nonlinear interactions, and provide data-driven, subject-specific models of excitability deployable within the temporal constraints of real-time pipelines.

In recent years, an increasing number of studies have begun to exploit ML/DL methods for the purpose to infer, from pre-stimulus EEG, the brain state that will determine the cortical or corticospinal response to an upcoming TMS pulse [19]. These studies can be broadly divided according to the outcome they aim to predict. Some focus on MEPs [17,32,36,79,81,82,83,84,85,86], i.e., corticospinal excitability, while a more limited number of studies targets TEPs [80,81,87], i.e., cortical excitability.

Methodologically, the MEP-prediction literature can be divided into two complementary strands. A first group of studies relies on feature-engineered EEG extraction, in which handcrafted spectral, temporal, or event-based descriptors (e.g., band-limited power, beta-burst characteristics, or oscillatory phase) are computed and fed into the predictive model [17,32,82,85,86]. The second group adopts data-driven decoding pipelines, in which spatial filters and discriminative features are learned directly from the EEG itself [79]. The prediction task is most often framed as a binary classification problem distinguishing high- from low-excitability trials, typically addressed using linear discriminant analysis (LDA) [82,85,86]. More comprehensive pipelines integrating sensor-, source-, and connectivity-level features have additionally employed ensemble classifiers such as Random Forest and linear Support Vector Machine (SVM) [17]. Other studies instead formulate the problem as a regression task [32,79]. Humaidan et al. [82,84], adopted a reinforcement-learning approach in which an agent learns online, without labelled data. Across the mentioned studies only two of them tested the pipeline online [82,86]. Reported performances across these studies converge within a relatively narrow range: classification accuracies between approximately 60% and 70% [19]. More in detail, reported classification performance ranges from an AUC of ~0.62 for LDA classifiers using power-spectral features [85], through 67–68% accuracy for logistic regression and regularized LDA pipelines [79] up to ~71% mean accuracy for the most comprehensive pipeline combining sensor-, source-, and connectivity-level features [17].

The prediction of TEPs from pre-stimulus EEG remains underexplored. A regression framework employed a nonlinear complexity measure (Higuchi’s Fractal Dimension), together with band-power features, to predict the evoked local field power following TMS; ensemble regressors explained approximately 70% of single-trial variance [87,88]. Using a complementary regression-based approach, fluctuations in pre-stimulus handcrafted features, including band-power features extracted from cortical parcels, together with coil position deviations and temporal trial trends, were shown to reliably predict TEP and MEP amplitudes [81]. The most advanced approach to date is PRIME, a deep learning architecture that predicts the N45 TEP component, a marker of excitation–inhibition balance, achieving a median ROC-AUC of approximately 0.68 [80]. Notably, the same model generalizes only partially to another TEP component (P60) and drops to near-chance performance when predicting MEPs, pointing to a partial dissociation between cortical and corticospinal excitability.

Several limitations of the reviewed studies warrant consideration. Sample sizes remain small (ranging from 8 to 50 participants across the reviewed studies), restricted to young, healthy volunteers; this dual constraint limits statistical power and raises concerns about generalizability to older or clinical populations, in whom oscillatory dynamics and cortical excitability profiles may differ substantially. Several studies further restrict their analyses to trials with extreme MEP or TEP values, selecting only the highest and lowest amplitude responses, to maximize class separability, which likely inflates apparent decoding performance [17,79,80,82]. Cross-subject generalization remains consistently poor, reinforcing the need for per-subject calibration and raising the question of whether population-level models will ever be viable outside single-subject adaptation schemes. Overfitting is a further concern: performance estimates obtained without nested cross-validation, or without a held-out test set entirely independent of feature selection, are likely optimistic, and direct comparison across studies is complicated by this heterogeneity in validation strategy.

A final, cross-cutting concern is interpretability: as ML/DL models grow in complexity, particularly DL architectures operating directly on raw EEG, their “black-box” nature [89] risks becoming a substantial barrier to clinical translation if interpretability is not incorporated by design. Progress toward explainability is therefore of equal importance to predictive performance in this domain: a systematic effort to link model decisions to established neurophysiological markers—oscillatory phase, power, or connectivity—will be essential to ensure that ML/DL-based brain-state definitions remain not only predictive, but also physiologically interpretable and clinically actionable, paving the way for the personalized, adaptive real-time frameworks. Despite these caveats, the trajectory of this literature outlines a concrete path toward fully adaptive, real-time brain-state-dependent EEG-TMS.

## 5. Discussion

### 5.1. Interpreting the Evidence

The studies reviewed here provide converging evidence that ongoing brain activity systematically shapes neuronal responsiveness to TMS. The phase of the sensorimotor mu-rhythm represents the first experimentally validated physiological marker of this relationship—one that has been successfully harnessed for real-time stimulation and replicated across multiple independent laboratories. However, the robustness of this evidence is not uniform: the clearest and most replicated results concern sensorimotor mu-rhythm-guided stimulation of M1 [1,43,53], while findings from non-motor regions [3,45], network-level states [15,35,41], and clinical populations [14,49,52] remain more limited and less consistent.

Importantly, the reproducibility of mu-rhythm-guided stimulation in the sensorimotor system has been facilitated by the unusually favorable characteristics of this experimental model, including a well-defined oscillatory generator, a high signal-to-noise ratio, and an objective physiological readout through motor-evoked potentials [1,53]. To date, replication has therefore been achieved primarily within this methodological framework, whereas findings from non-motor regions remains limited. Notably, several studies have reported modest or null effects despite successful technical implementation of brain-state-dependent stimulation [42,69]. Even among studies reporting significant group-level effects, substantial inter-individual variability remains [41,53,80]. Moreover, a recent meta-analysis suggests that neurophysiological effects are generally smaller and more heterogeneous than earlier literature implied [14].

These limitations collectively suggest that the gap between proof-of-concept and clinical utility is not primarily a matter of temporal precision, but of how comprehensively and individually brain states are characterized.

At present, the current literature is more informative about the feasibility of brain-state-dependent neuromodulation than about its clinical superiority over conventional stimulation paradigms [14,20]. The features that have made the sensorimotor system an ideal proving ground are largely absent in many neurological and psychiatric disorders. Whether the same principles remain effective when these favorable conditions are absent represents, therefore, the central question for the next research phase of the field. Chronic pain provides such a test case because it involves a distributed network dysfunction, lacks a single cortical target, and is characterized by substantial heterogeneity in treatment response [10,62,64]. Beyond chronic pain, adaptive EEG-TMS approaches have been explored in other clinical domains, including depression, OCD, and stroke rehabilitation, where early proof-of-concept findings suggest that brain-state-informed protocols may improve upon conventional open-loop paradigms [1,8,45,49]. However, the level of evidence remains highly variable across disorders—most convincingly demonstrated in motor stroke rehabilitation through mu-rhythm-guided stimulation [49], and considerably more preliminary in psychiatric conditions [14] where the relevant oscillatory targets, optimal stimulation sites, and physiological readouts are less well established.

Taken together, the evidence reviewed here suggests that the central challenge for the field is no longer to demonstrate that brain-state-dependent stimulation is feasible, but to determine how brain states should be defined to support robust and generalizable neuromodulation beyond the sensorimotor system. Whether richer representations combining source-level activity, network dynamics, and multivariate modelling can improve the prediction of stimulation responsiveness beyond local phase alone remains to be demonstrated. In this context, machine learning and deep learning approaches may ultimately play an important role by integrating high-dimensional neurophysiological features into individualized brain-state models suitable for adaptive neuromodulation [17,19,80]. These observations provide the rationale for the broader conceptual framework proposed in the following section.

### 5.2. Future Perspectives—Rethinking Brain States for Precision Neuromodulation

Despite the remarkable advances in brain-state-dependent stimulation, several conceptual and technical challenges must be overcome before its full potential can be realized--both as a therapeutic strategy for correcting maladaptive brain dynamics and as a precision tool for investigating causal brain-behavior relationships. Foremost among these challenges is the definition of the brain state itself. Whether the most informative representation is provided by local oscillatory phase, oscillatory power, functional connectivity, large-scale network dynamics, or multidimensional combinations of these features remains an open question.

Addressing this challenge will require moving beyond sensor-level representations toward more accurate and physiologically meaningful source-level estimates of ongoing brain activity. A promising direction is the development of individualized EEG-TMS frameworks that combine extensive offline characterization with efficient real-time monitoring. Structural MRI, diffusion MRI, and resting-state EEG can be analyzed without the computational constraints imposed by online operation to derive highly accurate forward models, individualized head conductivity models, source spaces, and subject-specific cortical regions of interest. In particular, EEG-TMS-tailored ROIs could be constructed by integrating information from multiple anatomical and functional cortical parcellations, all defined on the individual’s cortical mesh, thereby providing robust spatial priors for source reconstruction and brain-state estimation. Resting-state recordings could further be exploited to identify subject-specific oscillatory generators and functional networks, yielding personalized priors on the spatial, spectral, and connectivity properties of the relevant brain dynamics. These offline estimates would then inform the subsequent real-time experiment, allowing online algorithms to focus on tracking the instantaneous evolution of brain activity within an already optimized individualized model rather than repeatedly solving the inverse problem from scratch. Such hybrid offline–online frameworks have the potential to substantially improve the spatial specificity, physiological interpretability, and computational efficiency of brain-state estimation while remaining compatible with the stringent temporal requirements of closed-loop neuromodulation.

At the same time, the increasing complexity of brain-state estimation places stringent demands on the speed and reliability of online computations, necessitating efficient algorithms capable of extracting meaningful neural features with minimal latency. Overcoming these computational constraints will enable genuinely adaptive closed-loop protocols, in which stimulation parameters are continuously updated according to the evolving neural state. This, in turn, will pave the way for increasingly personalized interventions that account not only for an individual’s anatomy and physiology but also for the dynamic organization of their ongoing brain activity.

### 5.3. Translational Challenges and Clinical Implementation

Translation of brain-state-dependent neuromodulation into routine clinical practice will require addressing practical constraints in addition to demonstrating physiological efficacy. Current research implementations may involve substantial hardware and procedural complexity, including synchronized EEG–TMS acquisition, precise electrode localization, and, for accurate source-based approaches, high-density EEG and structural MRI for individualized anatomical modelling. These requirements increase cost, personnel demands, and workflow complexity, and may therefore limit direct transfer of research protocols to clinical settings. Importantly, however, not all components of an individualized closed-loop framework need to operate under real-time constraints. Computationally intensive procedures—including MRI-based head modelling, source-space construction, cortical parcellation, forward modelling, and calibration of patient-specific neural features—can be performed offline, using the extensive information available before the stimulation session. The online system can then be restricted to low-latency tracking of selected features within this precomputed individualized model, substantially reducing the computational burden of the real-time component. This offline–online architecture may therefore provide a pragmatic route toward clinical translation, although robust low-latency acquisition, EEG-TMS synchronization, artifact management, and reliable triggering remain essential.

Finally, reproducibility across institutions will require greater standardization of acquisition hardware, electrode montages, preprocessing, source-reconstruction procedures, state definitions, and decision thresholds. Such standardization should not necessarily imply a universal definition of brain state, but rather common procedures for calibration, validation, quality control, and reporting, allowing individualized state estimates to remain comparable across studies and clinical settings.

Ultimately, the central challenge is unlikely to be the identification of a single “optimal” brain state, but rather the development of increasingly accurate and individualized representations of the neural processes that determine responsiveness to stimulation. Future brain-state-dependent neuromodulation will therefore require the integration of multimodal neuroimaging, source imaging, machine learning, computational modeling, and adaptive control into a unified framework capable of continuously refining its estimate of the brain’s evolving state. Such systems would move beyond generic stimulation protocols toward selective modulation of dysfunctional network dynamics only when, where, and how intervention is required, preserving normal brain function while maximizing therapeutic efficacy. More broadly, this shift from fixed stimulation paradigms to adaptive, model-informed neuromodulation has the potential not only to improve clinical outcomes but also to establish causal brain stimulation as a precision tool for uncovering the dynamic principles that govern brain-behavior relationships.

## 6. Conclusions and Take-Home Messages

Over the past decade, brain-state-dependent neuromodulation has evolved from a conceptual hypothesis into a reproducible experimental framework demonstrating that both physiological and behavioral effects of non-invasive brain stimulation depend critically on the ongoing neural state. Accumulating evidence indicates that cortical excitability is not solely determined by stimulation parameters but is dynamically shaped by local oscillatory activity, large-scale network interactions, as well as individual anatomical and physiological factors. At the same time, these studies have revealed that brain states can hardly be reduced to a single EEG feature or universally defined across recording modalities. Rather, they should be regarded as operational constructs whose optimal representation depends on the scientific question, the measurement technique, and the computational model used to estimate it. The major challenge for the next decade is therefore unlikely to identify a single “optimal” brain state, but developing increasingly accurate, multimodal, and individualized models of brain dynamics capable of guiding adaptive neuromodulation with greater precision, reproducibility, and mechanistic specificity. Chronic pain, used here as a running example, illustrates both the promise and the size of the remaining gap: a network-defined, fluctuating disorder for which no local phase readout is sufficient, and for which the network-informed, brain-state-dependent framework outlined in Box 4 is now technically within reach.

In this sense, the evolution of brain-state-dependent neuromodulation is not simply a progression from open-loop to closed-loop stimulation, but from protocol-driven intervention toward model-driven control of brain dynamics.

Box 4 **Implementation of network-based real-time brain-state-dependent rTMS in pain research.**

Pain is a dynamic experience arising from coordinated activity across distributed cortical and subcortical networks [11], including sensorimotor, frontoparietal control, salience, and default mode networks [90]. Alterations within these systems have been implicated in the development and maintenance of chronic pain [62,63], consistent with emerging views that chronic pain reflects a disorder of large-scale network organization rather than localized dysfunction [64]. Therefore, chronic pain presents a clinically relevant test of whether brain-state-dependent stimulation can be extended to distributed and heterogeneous brain processes. Pain fluctuates over time and across individuals, reflecting dynamic changes in the underlying neural state that may contribute to variability in responses to non-invasive stimulation [12]. These features suggest that anatomical targeting alone may be insufficient and that stimulation effects may depend on the network state present at the time of delivery.

As neural oscillations are thought to coordinate communication within and between large-scale networks, they may provide an accessible electrophysiological mechanism through which maladaptive pain-related activity can be indexed and potentially modulated. Notably, recent multi-dataset evidence indicates that theta-band activity is the most consistently implicated frequency range across chronic pain conditions [91], suggesting a robust but non-specific potential biomarker of altered large-scale brain-state dynamics. Consistent with a functional role of theta in pain processing, disruptions of theta–gamma phase–amplitude coupling within the pain connectome have also been reported in chronic pain patients [92], suggesting that altered theta-mediated communication may contribute to pain persistence. One working hypothesis is that chronic pain involves dysregulated theta-mediated integration between internally oriented self-referential processing and salience-driven control systems. Such dysregulation could contribute to the persistence of pain-related cognition and affect.

Network-informed and brain-state-dependent approaches could therefore improve treatment precision by directly engaging the interacting systems that sustain chronic pain. In this context, theta-band connectivity may serve as a measurable index of large-scale network coordination, reflecting the interaction between posterior midline (default mode-related) and frontal midline (salience/control-related) systems. A potential real-time implementation would leverage source-space EEG connectivity following the framework of Marzetti et al. [36], which enables estimation of frequency-specific functional coupling while mitigating volume conduction effects. Although the accuracy of source-space reconstruction depends on methodological factors including individualized head models, adequate electrode density, and high signal-to-noise ratios, EEG signals can be reconstructed into cortical source space and aggregated into large-scale network-level regions of interest. Within this framework, theta-band connectivity can be quantified using phase-based metrics such as the weighted phase lag index (wPLI) or imaginary coherence, yielding a norm-referenced index of large-scale network integration along a frontal–posterior axis. Rather than assuming precise anatomical connectivity, this measure is interpreted as a systems-level state variable describing the degree of coupling between internally oriented and salience-related cortical dynamics. Deviations from healthy control distributions may therefore index maladaptive network states associated with chronic pain.

Importantly, emerging evidence suggests that theta-mediated network dynamics are not only measurable but can also be selectively engaged through brain-state-dependent stimulation. Gordon et al. [3] demonstrated that EEG-guided prefrontal rTMS delivered at different phases of ongoing theta oscillations modulates theta power and theta–gamma coupling. Although this study was not conducted in the context of pain neuromodulation, it provides proof of principle that theta-related brain states can be accessed and influenced in real time. On this basis, these stimulation approaches can be directly linked to theta-based network state variables derived from source-space EEG, including frontal–posterior theta connectivity and related indices of large-scale coupling. Extending this framework to chronic pain, such approaches may enable targeted interaction with aberrant theta-mediated network organization, with the goal of normalizing deviations in both theta-band connectivity and theta–gamma coupling identified at the network level. In the longer term, this approach could support the development of individualized neuromodulation strategies guided by patient-specific deviations in oscillatory network organization. Framed in this way, the proposal yields a directly testable prediction: rTMS delivered when frontal–posterior theta coupling deviates maximally from the normative range should produce greater normalization of that coupling—and greater analgesia—than the same protocol delivered at fixed intervals or during random network states. Crucially, each component of this pipeline (individualized source models, real-time LCMV beamforming, online connectivity estimation, and phase-specific prefrontal rTMS) has already been demonstrated in isolation; what remains is their integration and empirical test in a pain population.

## Figures and Tables

**Table 1:** Summary of real-time EEG-guided TMS studies. Overview of phase-dependent EEG-TMS studies detailing the triggering state, stimulated area, protocol, and principal findings. Green rows indicate repetitive TMS (rTMS) protocols; unshaded rows indicate single-pulse TMS (spTMS). Abbreviations: RMT, resting motor threshold; MEP, motor-evoked potential; TEP, TMS-evoked potential; LTP, long-term potentiation; LTD, long-term depression; SIHI, short-interval interhemispheric inhibition; iM1/cM1, ipsilesional/contralesional primary motor cortex.

Publication	N	Population	EEG Triggering State	Phase Estimation	Stimulated Area	TMS Pulse	Effect
Zrenner et al., 2018 [1]	12	Healthy, male, right-handed (26.5 ± 7.5 y)	Negative peak (high-excitability) vs. positive peak (low-excitability) of sensorimotor mu rhythm	AR forward prediction (order 30) of 8–12 Hz mu-signal	Left primary motor cortex (M1)	Single pulses and Repetitive triplets (3 pulses at 100 Hz, 80% RMT)	Negative peak spTMS led to larger MEPs than during positive peak. Negative peak rTMS led to LTP-like increases in MEP amplitude; positive peak or random phase produced no significant excitability changes.
Stefanou et al., 2018 [35]	16	Healthy right-handed volunteers	Negative peak mu rhythm (in-phase synchronization)	AR forward prediction (order 30) of mu-rhythm	Bilateral primary motor cortex (M1)	Dual-coil paired pulses (SIHI protocol)	Strongest short-interval interhemispheric inhibition (SIHI) when both M1 sites were in-phase for the mu-rhythm negative peak.
Desideri et al., 2019 [42]	12	Healthy (4 M), right-handed (27.5 ± 7.7 y)	Negative peak of sensorimotor mu rhythm	Real-time AR-based prediction	Left primary motor cortex (M1)	Single pulses at 90% or 110% RMT	Higher absolute amplitudes of MEPs (at 110%) and TEPs (P70/N100) compared to positive peak targeting.
Baur et al., 2020 [2]	12	Healthy young adults (24.2 ± 3.3 y)	Negative peak vs. positive peak vs. random phase of sensorimotor mu rhythm	Real-time AR-based	Left primary motor cortex (M1) (hand area)	1 Hz rTMS (900 pulses, 110% RMT)	Positive peak induced significant LTD-like MEP reduction; negative peak showed a trend toward LTP-like plasticity; random phase showed non-significant LTD trend.
Stefanou et al., 2020 [43]	51	Healthy (29 F), right-handed (24 ± 6 y)	Negative peak vs. positive peak of mu rhythm (8–13 Hz)	AR forward prediction (order 30)	Bilateral primary motor cortex (M1) (hand areas)	Single-pulse TMS	Lowest Resting Motor Threshold (RMT) observed at the negative peak; highest RMT observed at the positive peak in both hemispheres.
Torrecillos et al., 2020 [44]	17	Healthy volunteers (10 F) (35.3 ± 13 y)	Optimal phase of beta oscillation (realigned peaks)	Fourier transform on 2 cycles preceding TMS	Primary motor cortex (M1)	Single-pulse TMS (120% RMT)	Targeted beta phase stimulation resulted in greater MEP amplitude, lower coefficient of variation, and shorter onset latency.
B. Zrenner et al., 2020 [45]	17	Patients with Major Depressive Disorder	Negative peak of instantaneous alpha oscillations (8–12 Hz)	Real-time AR prediction of DLPFC alpha (Hjorth-F5)	Left dorsolateral prefrontal cortex (DLPFC)	Repetitive triplets (3 pulses at 100 Hz, 70% RMT)	Alpha-synchronized rTMS reduced resting-state alpha power and increased TMS-induced beta oscillations; iTBS and random-phase stimulation did not.
Baur et al., 2022 [46]	12	Healthy (25.2 ± 3.7 y)	Trough of sensorimotor mu rhythm vs. random phase	Mu trough triggered quadruple bursts	Left primary motor cortex (M1)	200 quadruplet bursts at 100 Hz or 200 Hz	Mu-trough stimulation (100/200 Hz) induced LTP-like MEP increases; random-phase stimulation (200 Hz) induced LTD-like MEP decreases.
Gordon et al., 2022 [3]	16	Healthy adults (23.4 ± 3.2 y)	Negative peak vs. positive peak of prefrontal theta oscillation (4–8 Hz)	Real-time source-based beamforming for DMPFC theta	Left dorsomedial prefrontal cortex (DMPFC)	Repetitive (100 Hz) triplet bursts at 120% RMT	Negative peak: Increased TMS-induced theta power, theta–gamma phase–amplitude coupling, and decreased working memory response time. Positive peak: Decreased theta power.
Momi et al., 2022 [47]	20	Healthy volunteers (two visits)	Negative peak (trough) vs. positive peak of sensorimotor mu rhythm (8–13 Hz)	Laplacian filter on C3; individual mu-peak	Left primary motor cortex (M1)	Single pulses (120% RMT)	Mu-trough stimulation induced significantly higher inter-hemispheric phase-lock synchronization (M1-M1) in the mu band compared to peak stimulation.
Wischnewski et al., 2022 [48]	20	Healthy (11 F) (22.7 ± 2.9 y)	Mu trough (180°) vs. Beta peak (0°) and associated phases	Educated Temporal Prediction (ETP) using training data	Left primary motor cortex (M1)	Suprathreshold single-pulse TMS	Maximal MEPs at mu trough (180°) and rising phase (90°); maximal MEPs at beta peak (0°) and falling phase (270°); rhythms show opposing excitability patterns.
Vetter et al., 2023 [41]	15	Healthy, right-handed (23.8 ± 2.4 y)	High vs. low functional connectivity (stPLV) between bilateral motor cortex	Real-time functional connectivity (stPLV)	Left primary motor cortex (M1)	Biphasic single pulses (110% RMT)	MEP amplitudes were significantly larger during high interhemispheric functional connectivity states compared to low connectivity states.
Mahmoud et al., 2024 [49]	30	Chronic stroke patients (15 per group)	Trough of ipsilesional sensorimotor mu rhythm	Real-time AR-based (BOSS device) mu trough	Ipsilesional primary motor cortex (M1)	Repetitive triplets (400 triplets at 100 Hz, 1200 pulses) at 100% RMT	Significant improvement in motor impairment and function, and objective reduction in spasticity in stroke patients.
Perera et al., 2024 [50]	34	Healthy adults (23.6 ± 2.0 y)	Trough and falling phases of mu oscillation (8–13 Hz)	AR-based prediction for subthreshold (90% RMT)	Primary motor cortex (M1)	Single-pulse TMS (120% RMT)	Increased modulation of the early cortical response, specifically the P50-N15 TEP complex.
Brancaccio et al., 2025 [51]	19	Chronic stroke patients	Trough (high-excitability) vs. no-trough (low-excitability) of sensorimotor mu-rhythm	Post hoc AR reconstruction of phase	Ipsilesional (iM1) and contralesional (cM1) primary motor cortex	Single-pulse TMS (115% RMT)	Differentiation in cM1 showed larger TEPs at trough; differentiation in iM1 was significant only for post-pulse beta power, which correlated with individual motor function
Wischnewski et al., 2025 [52]	11	Chronic stroke survivors	Peak, fall, trough, and rise in the sensorimotor mu-oscillation (8–13 Hz)	ETP algorithm	Primary motor cortex (M1) in affected and unaffected hemispheres	Biphasic single-pulse TMS (typically at 120% RMT)	MEPs were increased at trough and decreased at peak. Notably, phase modulation strength diminished in patients with more severe motor impairment, and TEP phase preference was abolished in the stroke-affected hemisphere

**Table 2:** From current evidence to clinical translation: unresolved challenges and validation requirements for brain-state-dependent neuromodulation.

Dimension	Current Evidence	Unresolved Problem	Needed Validation	Clinical Translation Criteria
Local oscillatory phase	Phase-dependent stimulation can modulate physiological and behavioral responses, particularly for sensorimotor rhythms.	Effects are heterogeneous and highly dependent on oscillatory target, recording method, and stimulation protocol; local phase may provide only a partial representation of brain state.	Replication across regions, frequencies, tasks, and independent cohorts; prospective prediction of stimulation response.	Robust subject-specific phase estimation; reproducible effects; clinically meaningful benefit over non-state-dependent stimulation.
Individualized brain-state estimation	Individual anatomy, physiology, and baseline neural activity influence responsiveness to stimulation.	No consensus exists on which features best define an individual’s relevant stimulation state.	Prospective testing of individualized predictors against predefined outcomes; test–retest reliability and cross-session stability.	Automated calibration, clinically feasible acquisition, reproducible state estimates, and acceptable setup time.
Source-level estimation	Source reconstruction can reduce sensor-level spatial mixing and provide anatomically constrained estimates of cortical activity.	Accuracy depends on head modelling, electrode registration, SNR, preprocessing, and inverse modelling assumptions.	Validation against independent measures and assessment of robustness to modelling and recording variability.	Reliable performance with clinically feasible EEG configurations and computational requirements.
Network/connectivity states	Inter-regional connectivity can predict responsiveness to stimulation and may provide information not captured by local oscillatory phase.	Connectivity metrics are not intrinsically periodic; optimal thresholds, temporal windows, and relevant network configurations remain unclear.	Prospective threshold-based triggering; characterization of trigger-rate stability and prediction of plasticity or behavioral outcomes.	Reliable detection at clinically feasible trigger rates, with subject-specific calibration and robust false-trigger control.
Multidimensional brain states	Combining local oscillations, connectivity, anatomy, and other physiological features may provide a richer representation of responsiveness.	More features increase model complexity, risk of overfitting, and computational demands.	Out-of-sample and prospective validation against simpler models; ablation studies to determine which features provide incremental predictive value.	Parsimonious models with demonstrable benefit, low latency, robustness, and interpretable decision rules.
Machine learning	ML can integrate multiple neural features and identify nonlinear relationships between pre-stimulation state and stimulation response.	Generalizability, interpretability, dataset size, and susceptibility to overfitting remain major concerns.	Independent-cohort validation, preregistered prospective prediction, transparent feature reporting, and comparison with simple baselines.	Explainable and robust models that generalize across sessions, subjects, and sites.
Offline–online integration	MRI, resting EEG, and other measurements can provide individualized priors before the real-time experiment, reducing the computational burden of online estimation.	Optimal division between offline modelling and online adaptation remains to be established.	Direct comparison of offline-informed versus purely online approaches; assessment of robustness to changes in brain state and electrode configuration.	Short preparation time, automated calibration, reliable real-time operation, and compatibility with existing clinical workflows.
Standardization and reproducibility	Closed-loop studies currently use heterogeneous hardware, preprocessing pipelines, state definitions, and stimulation protocols.	Lack of common standards limits comparison and replication across laboratories.	Harmonized acquisition/reporting standards, quality-control procedures, benchmark datasets, and multicenter validation.	Reproducible performance across sites, operators, hardware platforms,

## Data Availability

No new data were created or analyzed in this study. Data sharing is not applicable to this article.

## Abbreviations

The following abbreviations are used in this manuscript:

BOLD	Blood Oxygenation Level-Dependent
ccPAS	Cortico-Cortical Paired Associative Stimulation
DL	Deep Learning
DLPFC	Dorsolateral Prefrontal Cortex
DMPFC	Dorsomedial Prefrontal Cortex
EEG	Electroencephalography
fMRI	Functional Magnetic Resonance Imaging
iTBS	Intermittent Theta-Burst Stimulation
LCMV	Linearly Constrained Minimum Variance
LDA	Linear Discriminant Analysis
LTD	Long-Term Depression
LTP	Long-Term Potentiation
M1	Primary Motor Cortex (iM1/cM1: ipsilesional/contralesional M1)
MDD	Major Depressive Disorder
MEG	Magnetoencephalography
MEP	Motor-Evoked Potential
ML	Machine Learning
MRI	Magnetic Resonance Imaging
NIBS	Non-Invasive Brain Stimulation
OCD	Obsessive–Compulsive Disorder
PET	Positron Emission Tomography
PRIME	Predictive Recurrent Inference for Motor Excitability
RMT	Resting Motor Threshold
ROC-AUC	Receiver Operating Characteristic—Area Under the Curve
ROI	Region of Interest
rTMS	Repetitive Transcranial Magnetic Stimulation
S4	Structured State-Space Modeling
SEEG	Stereoelectroencephalography (Stereo-EEG)
SIHI	Short-Interval Interhemispheric Inhibition
SNR	Signal-to-Noise Ratio
spTMS	Single-Pulse Transcranial Magnetic Stimulation
stPLV	Short-Term Phase-Locking Value
